# Government funding of research beyond biomedicine: challenges and opportunities for neuroethology

**DOI:** 10.1007/s00359-022-01552-3

**Published:** 2022-05-10

**Authors:** Günther K. H. Zupanc, Wolfgang Rössler

**Affiliations:** 1 Department of Biology, Northeastern University, Boston, MA 02115 USA; 2 Behavioral Physiology and Sociobiology (Zoology II), Biocenter, University of Würzburg, 97074 Würzburg, Germany

**Keywords:** German Research Foundation, Government research funding, National Science Foundation, Deutsche Forschungsgemeinschaft, Neuroethology

## Abstract

Curiosity-driven research is fundamental for neuroethology and depends crucially on governmental funding. Here, we highlight similarities and differences in funding of curiosity-driven research across countries by comparing two major funding agencies—the National Science Foundation (NSF) in the United States and the German Research Foundation (*Deutsche Forschungsgemeinschaft*, DFG). We interviewed representatives from each of the two agencies, focusing on general funding trends, levels of young investigator support, career-life balance, and international collaborations. While our analysis revealed a negative trend in NSF funding of biological research, including curiosity-driven research, German researchers in these areas have benefited from a robust positive trend in DFG funding. The main reason for the decrease in curiosity-driven research in the US is that the NSF has only partially been able to compensate for the funding gap resulting from the National Institutes of Health restricting their support to biomedical research using select model organisms. Notwithstanding some differences in funding programs, particularly those relevant for scientists in the postdoctoral phase, both the NSF and DFG clearly support curiosity-driven research.

## Introduction

In 1973, Karl von Frisch, the founder of this journal, received together with Konrad Lorenz and Niko Tinbergen the Nobel Prize in Physiology or Medicine for their pioneering work on animal behavior. Both von Frisch and Lorenz were sons of privilege. Based on the wealth of their families, they were able to endure times of unemployment, political oppression, or war, and support their own research (Nisbett 1976; Krebs and Sjölander 1992; Munz 2016).

Most scientists today are less privileged. They need funding for their research and often, especially during early stages of their career, for their own positions. This need is particularly critical in areas like neuroethology, where no adequate job perspectives exist outside of academia. Like in other basic science areas, the major source of financial support for neuroethological research is governmental grants, thus underscoring the importance of public funding for this discipline.

In the United States, a major shift in governmental funding of neuroethological studies took place around the turn of the millennium, particularly after the National Institutes of Health (NIH) in 1999 published a list of officially recognized model organisms for biomedical research, which ultimately consisted of 13 species, including mouse (*Mus musculus*), rat (*Rattus norvegicus*), domestic chicken (*Gallus gallus*), African clawed frog (*Xenopus laevis*), zebrafish (*Danio rerio*), fruit fly (*Drosophila melanogaster*), and roundworm (*Caenorhabditis elegans*) (Ankeny and Leonelli 2020; Farris 2020). These organisms had originally been chosen due to practical considerations, such as ease of housing and breeding in the laboratory, high fecundity, short generation time, and genetic homogeneity, rather than as optimal fits for answering specific questions related to human disease (Bolker 2012). Nevertheless, the sequencing of their genomes (*C. elegans*: 1998; *D. melanogaster*: 2000; *M. musculus*: 2002; *G. gallus*: 2004; *R. norvegicus*: 2004; *D. rerio*: 2013; *X. laevis*: 2016) enhanced their utility as models for human disease (Green et al. 2011). This focus on a select few genetic model systems was consistent with the launch, in 2004, of a goal-driven investment strategy known as the NIH Roadmap for Medical Research. It consisted of a set of programs designed to transform medical research capabilities and speed the movement of research from the laboratory to the patient’s bedside, emphasizing the importance of discoveries directly related to diseases (National Institutes of Health 2014). The focus on genetic model organisms, as well as research directly related to human disease, has made it increasingly difficult to justify, and thereby obtain, funding from NIH for curiosity-driven basic research, including research in neuroscience and neuroethology (Bolker 2012; Brenowitz and Zakon 2015; Yartsev 2017; Farris 2020).

To obtain a better picture of the funding situation for neuroethology in recent years, we first collected and analyzed publicly available data on federal funding in the United States because such information can be readily extracted from publicly available databases. We then discussed our analysis with representatives of two major funding organizations that support a significant portion of neuroethological research—the National Science Foundation in the United States (NSF; Box 1) and the *Deutsche Forschungsgemeinschaft* (German Research Foundation) in Germany (DFG; Box 2). Based on the number of papers published in the *Journal of Comparative Physiology A*, these two countries are prime contributors to the global research output in this discipline. Between 2017 and 2019, 114 of 244 published articles (equivalent to 47%) originated from the United States and Germany.

**Figure:**
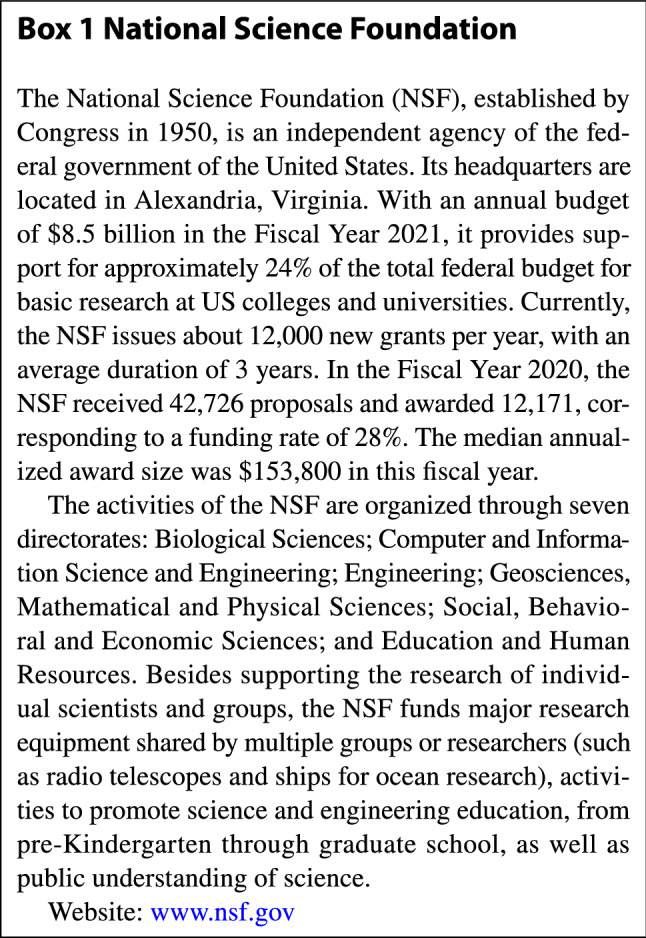

**Figure:**
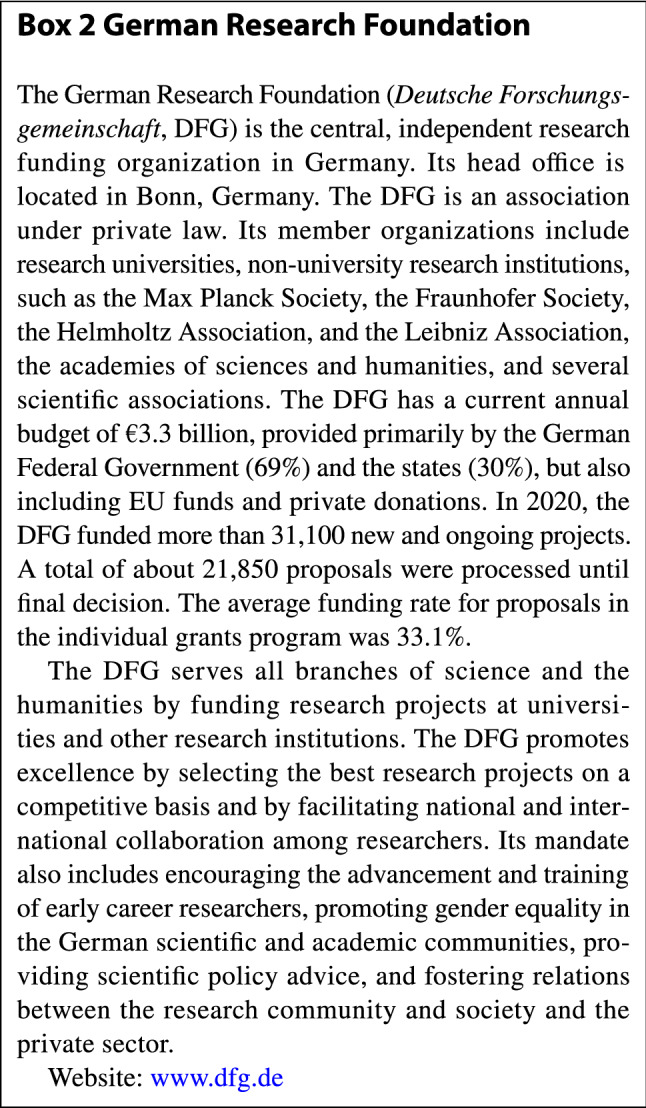

## Governmental funding of neuroethology: an analysis of the situation in the United States

**Fig. 1:**
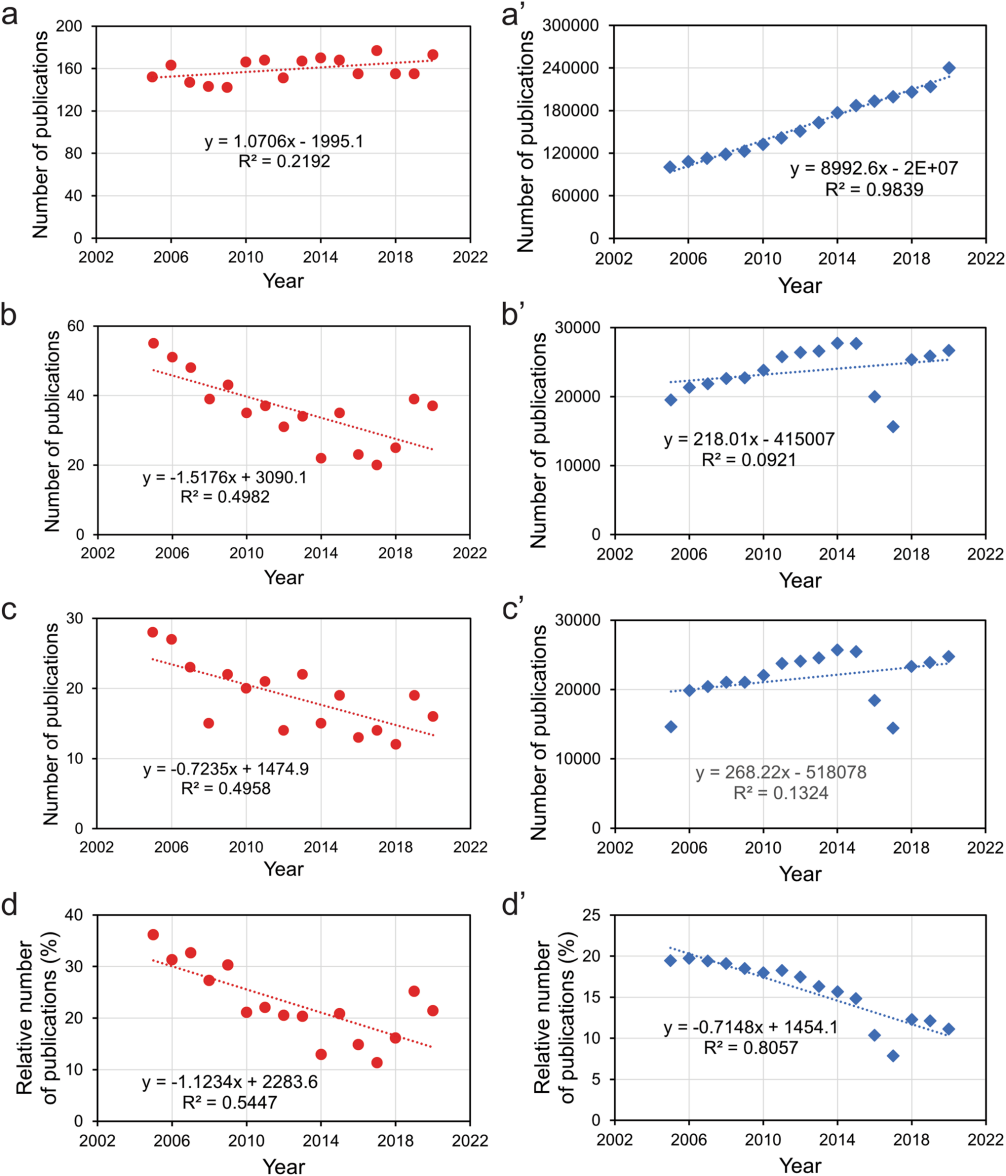
Publication output of research in neuroethology (**a**–**d**) and neuroscience (**a**’–**d**’) between 2005 and 2020. Representative samples of publications were retrieved from the PubMed database using the queries ‘neuroethol*’ and ‘neuro*,’ respectively. **a**, **a**’ Total number of journal articles published. **b**, **b**’ Number of journal articles that had reported US Government research support. **c**, **c**’ Number of journal articles that had indicated (intramural and extramural) NIH support. **d**, **d**’ Percentage of journal articles that had reported research support by the US Government among the total number of publications in the respective sample. The dotted lines represent the fitted linear regression curves

For our initial analysis of the funding situation in neuroethology, we searched PubMed (https://pubmed.ncbi.nlm.nih.gov/) for papers with publication dates between 2005 and 2020 using the term ‘neuroethol*’ in all fields. This query retrieved publications containing the terms ‘neuroethology,’ ‘neuroethological,’ and ‘neuroethologist.’ Although these papers clearly reflected only a sample of the whole neuroethological literature published in a given year, we assumed that the relative size of this sample did not differ substantially over the years. On the other hand, inspection of a random selection of the publications retrieved using this query confirmed that the research focus of most of them was, indeed, neuroethology. Analysis of the annual number of publications indicated a slight, but not significant, increase over the past 16 years (Fig. 1a).

The results of our search were then filtered for those papers that had indicated research support by the US Government. Over the observed time frame, the trend revealed a significant decline in the number of neuroethological papers that reported US-Government funding (Fig. 1b). Approximately half of this decline can be attributed to the decrease in the number of publications that were based on extramural or intramural funding by the National Institutes of Health (NIH) (Fig. 1c). To normalize these data, we calculated the relative number of papers that stated US-Government funding, relative to the total size of our sample of neuroethological papers published in a given year. A plot of these data suggests that the fraction of neuroethological studies that received US-Government funding in 2020 was cut in half, compared to 2005 (Fig. 1d).

Is this decline in US-Government funding of neuroethology (as indicated by publications) due to an issue specific to this discipline, or is it a wider problem of neuroscience research in general? To address this question, we searched in the PubMed database for papers published between 2005 and 2020, using the term ‘neuro*’. This search strategy retrieved publications in a wide range of basic and clinical neuroscience disciplines. Over this time period, the total number of publications in the neurosciences increased dramatically, from approximately 100,000 in 2005 to over 240,000 in 2020 (Fig. 1a’).

Of these publications, papers that indicated US-Government support did not show a significant (*p* > 0.05) trend over time (Fig. 1b’), and neither did papers that reported extramural or intramural NIH funding (Fig. 1c’). However, normalization of these data revealed that the contribution of scientific publications resulting from US-Government funding, relative to the total number of neuroscience papers, declined significantly by roughly 50% over the same time period (Fig. 1d’). Thus, the decline in US-Government-funded studies in neuroethology shows a similar trend to the neurosciences in general.

Our analysis suggests that a major factor in the decline of publication output resulting from research funded by the US Government is the continued reduction in support by the NIH, beyond their initial shift in funding priorities around the turn of the millennium. An obvious question to ask is whether funding by the NSF has been able to compensate for this loss in NIH funding opportunities. Since data are not publicly available for proposals in the area of neuroethology, we evaluated various indicators of funding for proposals submitted to the Directorate for Biological Sciences of this agency, using the online tool ‘NSF By The Numbers’ (https://tableau.external.nsf.gov/views/NSFbyNumbers/Trends?%3AisGuestRedirectFromVizportal=y&%3Aembed=y&%3Alinktarget=_blank&%3Atoolbar=top). This tool provides statistical information for the last 10 years, from 2012 to 2021.

**Fig. 2:**
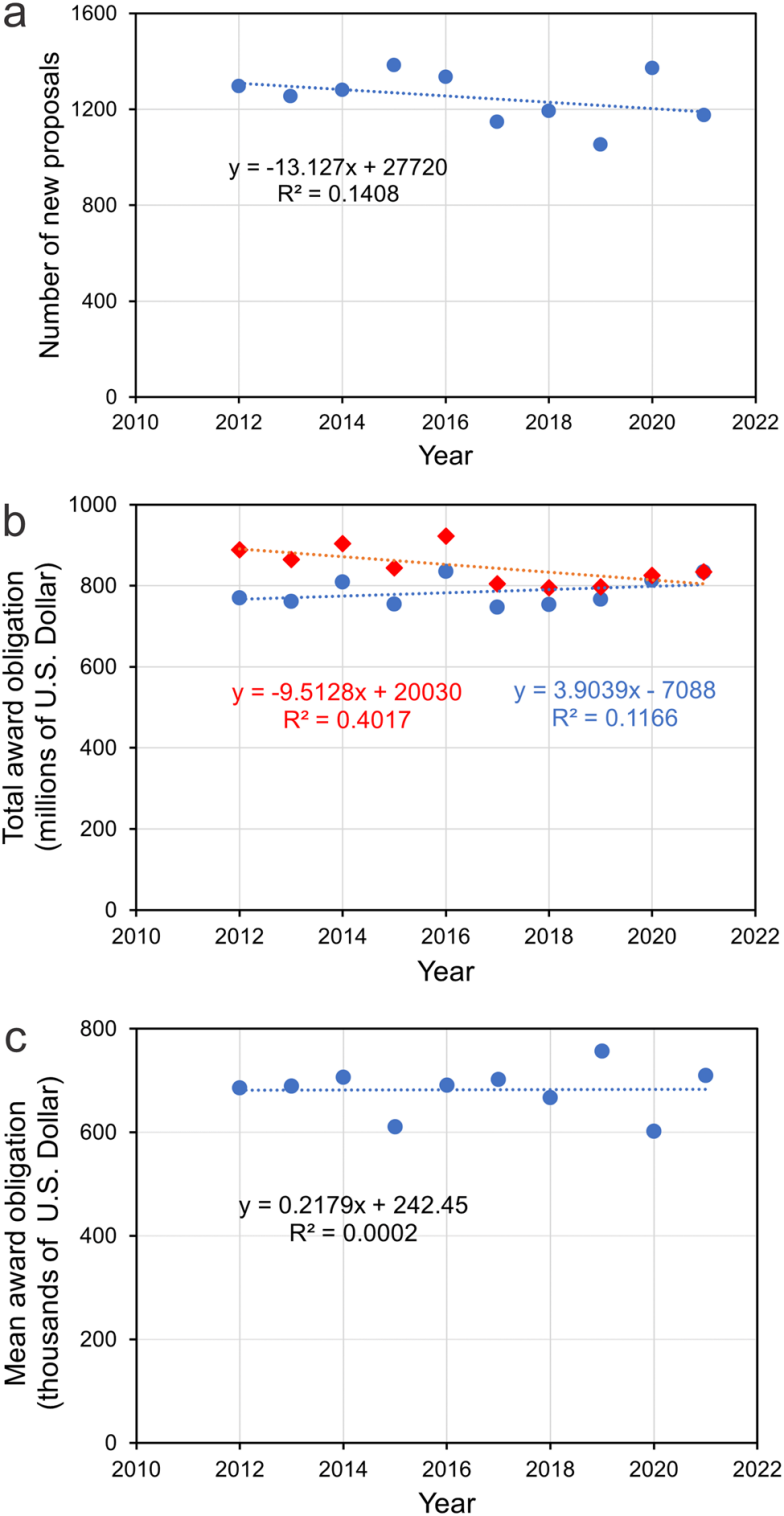
Funding of biological research by the National Science Foundation between 2012 and 2021. **a** Number of new proposals funded. **b** Total award obligations for funding biological research proposals in US Dollars (blue filled circles) and inflation-adjusted US Dollars (red filled diamonds). **c** Mean award obligation for funding biological research proposals in inflation-adjusted US Dollars. The dotted lines represent the fitted linear regression curves

Comparison of the number of new awards (Fig. 2a), and the total award obligation (Fig. 2b; *blue trendline*), failed to reveal a significant (*p* > 0.05) trend between 2012 and 2021. After conversion of these amounts to inflation-adjusted US Dollars using the CPI Inflation Calculator of the United States Bureau of Labor Statistics (https://data.bls.gov/cgi-bin/cpicalc.pl), regression analysis indicated a significant (*p* < 0.05) decrease in the total award obligation by approximately 10% over the last 10 years (Fig. 2b; *red trendline*). However, translation of these data into mean inflation-adjusted award obligations showed no significant (*p* > 0.05) change over the last 10 years (Fig. 2c). Assuming that funding of neuroethological research exhibits a similar trend as biological research in general, we conclude that the number of proposals funded by NSF has decreased over the last 10 years by approximately 10%, while the mean amount awarded (adjusted for inflation) has remained largely unchanged over this time period.

## Beyond the data: funding neuroethology by NSF and DFG

**Fig. 3:**
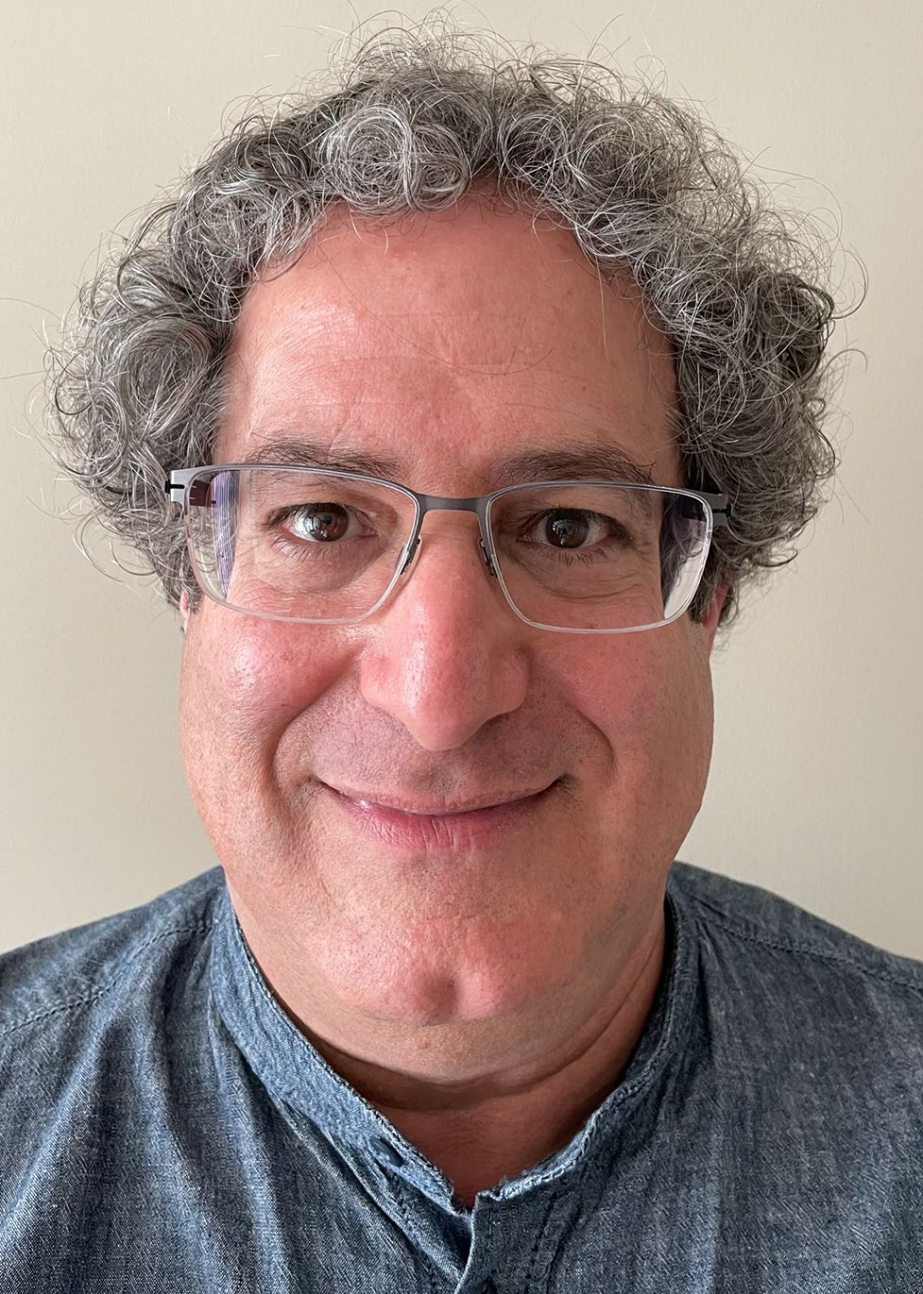
Evan Balaban, PhD, is Program Director of the Neural Systems Cluster, Division of Integrative Organismal Systems in the Biology Directorate of the National Science Foundation in Alexandria, VA (Washington, DC). (Photograph by M. Pompeiano.)

**Fig. 4:**
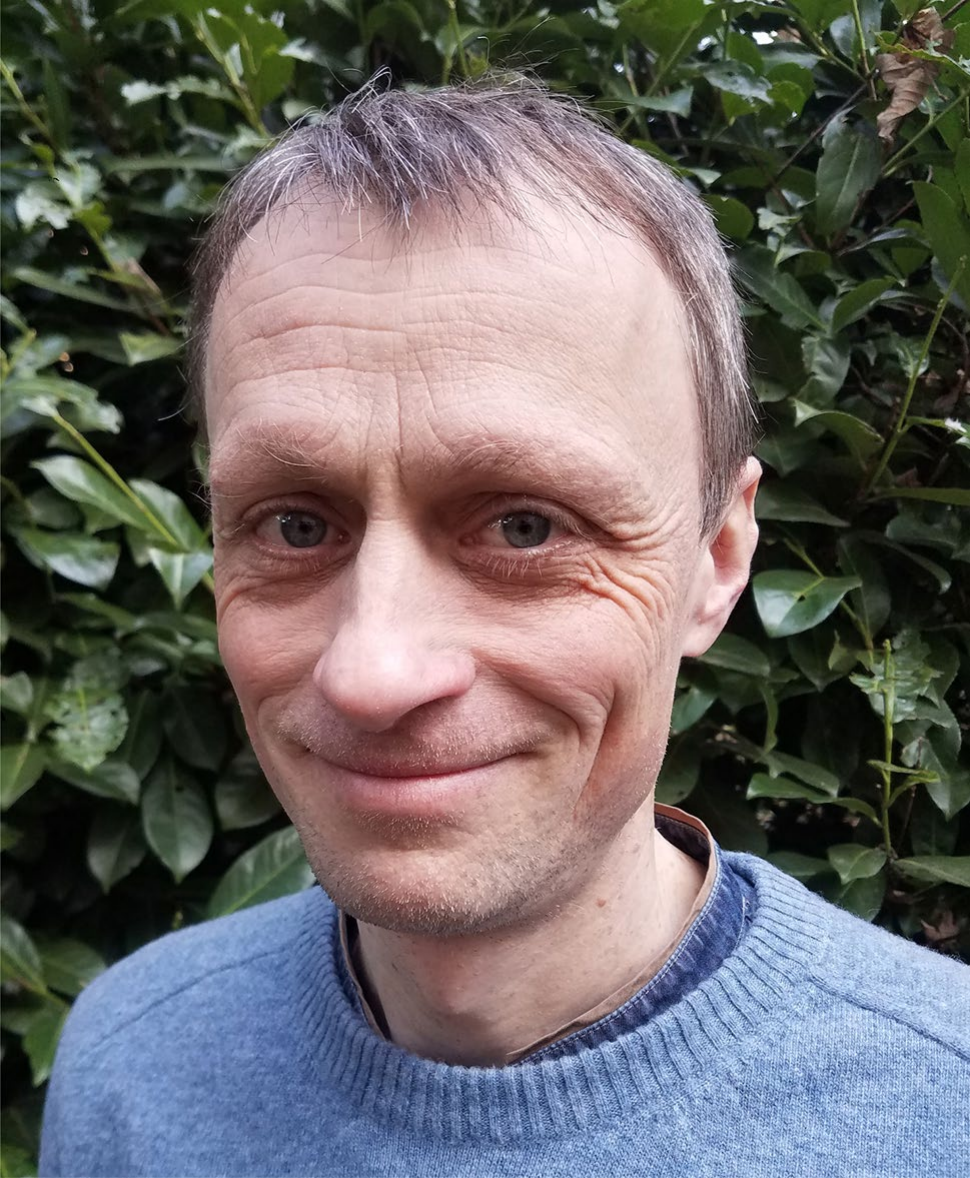
Dr. Christoph Limbach is Program Director in Neuroscience at the Head Office of the *Deutsche Forschungsgemeinschaft* (German Research Foundation) in Bonn, Germany. (Photograph by F. Limbach.)

To discuss our analysis of the funding of neuroethology, we met online with Evan Balaban (Fig. 3) as representative of the NSF and Christoph Limbach (Fig. 4) as representative of the DFG. In addition to their interpretation of the overall picture, we were especially interested in learning about opportunities for funding by these two organizations of students, postdocs, and young independent investigators, and about their efforts to support career-life balance and international collaborations.

### “We love to receive projects on any species!”—the role of model organisms and organismal diversity in research funding

*Günther K.H. Zupanc:* Our analysis indicates a significant decline over the last 16 years in output, as measured by the number of publications, of research funded by the US Government in neuroscience in general and in neuroethology in particular. According to your observations, are the result of our analysis a fair reflection of the situation of funded research in the United States?

*Evan Balaban:* I think I can answer this question best from my own personal experience as an active researcher in neuroethology before I joined NSF in 2015. Drawing from this experience, I know that people familiar with the subject would likely come to a similar conclusion as you did. In my opinion, the important question is about the driving forces behind the numbers. To answer this question let me produce some context. 30 years ago, both the NSF and the NIH funded basic research using a wide range of species and a wide range of questions. At that time there were more neuroethology proposals funded overall than there are today. As NIH began to focus more on projects using model organisms with well-developed genetic resources that addressed issues more directly related to human diseases, people working in research areas no longer supported by NIH applied in larger numbers to NSF, which continued to support non-disease-related work on any organism that answered important basic science questions. The initial decade of the 2000s was characterized by an increase in grant applications to NSF in areas like neuroethology.

*Günther K.H. Zupanc:* So, it was the announcement by NIH of their list of recognized model organisms in 1999 that triggered this fundamental shift?

*Evan Balaban:* I would agree that this was an important factor, but this was not just about the organisms. It was also about the disease-related focus of the questions being posed. Before that, NIH funding programs were supporting a broad range of basic-research questions in a broad range of species. This became more restricted with respect to both what species were utilized, and how directly relatable to diseases the aims of projects were. More recently, NIH has been broadening their funding of both species and scientific topics, but this is still not as broad as it once was. As a result of these historical changes in NIH funding priorities in areas such as neuroethology, there was an increase in grant submissions to NSF (without a corresponding increase in the governmental funding allocations to NSF). This effectively meant that a greater number of proposals were now competing for less total federal dollars than before.

*Günther K.H. Zupanc:* How did NSF respond to this sudden increase in the number of proposals submitted?

*Evan Balaban:* I can answer this question best from my perspective as an NSF program officer. Even though NSF had two submission deadlines for full grant proposals each year, this system rapidly became administratively difficult, because of the sheer number of grant reviews that it required. It became very hard to get enough reviewers to support this system because we had to ask people so frequently for reviews. NSF introduced a pre-proposal system to deal with these issues. People first submitted a four-page proposal that went through an initial cycle of review, and then we invited a proportion of those investigators to submit a full proposal. However, our scientific communities disliked this system, because it reduced the rate at which investigators could apply for grants. In response to these concerns, the NSF Biology Directorate switched to a new system. There are now no proposal submission deadlines, and people can submit a proposal at any time. Other directorates at NSF tried this system out before Biology adopted it and found that it initially decreased the overall number of proposals, and that the apparent ‘quality’ of the proposals (as judged by reviewer evaluations) went up. After the introduction of the new system, the number of proposals submitted did decrease, but now this number is back up to the number of full proposals we were receiving previously. This is part of the story that the graphs are showing.

Another factor that is missing from the analysis is that the distribution of award sizes has changed. In the past, we were getting quite a wide distribution of budgets for projects. Now, budgets are skewed to larger and larger amounts. It just takes more money to do research, especially if one carries out mechanistic studies and is trying to use state-of-the art techniques. These costs have skyrocketed. What that means is that we are not able to support as many projects as we did in the past because most of them are expensive.

So, it is a combination of two major factors that have led to a decline in US federal support available for neuroethology research: more proposals are competing with each other for an overall smaller share of federal dollars, and the cost per proposal has been going up. Although NSF annual budgets have been increasing, historically those increases have not been sufficient to offset these historical trends.

*Günther K.H. Zupanc:* You mentioned the importance of model organisms in the decision made by NIH to withdraw from supporting neuroethological research. Does the choice of organisms play any role when proposals are evaluated by NSF?

*Evan Balaban:* To answer your question, let me refer to our synopsis of the mission of the Neural Systems Cluster. We explicitly encourage the use of comparative approaches, studies of organisms in their natural habitat, and the development of novel theoretical, computational, and transdisciplinary approaches to guide and instruct experimental design. So, we clearly say we would like investigators to be doing comparative work. What we mean by this is: we love to receive projects on any species!

On the other hand, if an investigator is using a so-called model species but is asking an original and interesting neuroethological question, we are happy to consider that work. We would not exclude investigators because they are working on a model species.

We are especially interested in encouraging studies on species for which not a lot is previously known. For instance, a few years ago we initiated a program called Enabling Discovery through GEnomic Tools (EDGE). The goal of this program is to help fund people who want to establish genetic approaches using modern tools like CRISPR-Cas9 technology to start doing genetic work on non-traditional or non-model species. The idea is to try to spread the use of more modern technology among our traditional disciplinary areas—and neuroethology is certainly one of those that is uppermost in our minds when we think of these things. So, yes, we are very, very interested in encouraging people to study whatever species they happen to be studying.

*Wolfgang Rössler:* I am not aware of any restrictions in terms of model organisms, like the ones implemented in the policy of NIH, at DFG. Is that, indeed, the case?

*Christoph Limbach:* Yes, this is the case. We do not have any restrictions in regard to model organisms. It is up to the researchers themselves to design their research projects and choose the model organism that is best suited. In the past, the Neurosciences Review Board of the DFG has repeatedly emphasized that we need diversity among experimental models, and that contributions from studies on non-model organisms are highly valuable. Thus, DFG is truly open to such studies.

*Wolfgang Rössler:* The published DFG budget figures suggest an overall increase in the budget between 2016 and 2020 for the Review Boards Zoology (which includes subject area 203-04, ‘Sensory Biology and Behavior’) and Neurosciences (which includes subject area 206-04, ‘Systems, Computational and Behavioral Neuroscience’). Most neuroethology proposals are evaluated by these two Review Boards. Do these numbers reflect an increase in the total numbers of grants awarded, or have projects simply become more expensive, as Evan had mentioned for NSF grants?

*Christoph Limbach:* Both are true to a certain extent. First of all, DFG is extremely grateful for a steady annual increase of its budget, now over more than a decade. We greatly appreciate the strong commitment of the German Federal Government to basic science. At the same time, we observe a constant increase in the number of proposals submitted to the DFG.

For our discussion, it is important to understand that we do not allocate a specific budget to certain subject areas. Thus, we do not set certain funding priorities. Instead, we apply an algorithm to assign the available total budget to the different subject areas. This algorithm reflects the demand for funding within each subject area and takes into account the requested budget in the preceding year as well as the approved budget over the past 2 years. So, an increase in the budget that exceeds the average reflects an increasing demand in this subject area.

The other effect, increases in the budget awarded per proposal, is less relevant for individual grants (here this effect is mostly linked to inflation and increases in staff expenses). However, such increases in the budget are highly relevant in some other DFG programs. For example, DFG’s Junior Research Groups in the Emmy Noether program are now funded for 6 years, instead of 5 years previously, and the funding period for Research Units (*Forschungsgruppen*) has been extended to two periods of 4 years.

### Supporting undergraduate researchers: not just for washing dishes

*Günther K.H. Zupanc:* In the second part of our conversation, we will focus on specific aspects of funding of neuroethological research, especially during early career stages. As part of this discussion, we would also like to learn more about the similarities of, and the differences between, the funding mechanisms of the NSF and the DFG. Let’s start with support of undergraduate research. At the NSF, the major funding instruments are the Research Experiences for Undergraduates (REU) programs. Besides promoting excellence in teaching, they were specifically designed to encourage undergraduate research. How do these programs work?

*Evan Balaban:* NSF has an entire Directorate for Education and Human Resources. The administered programs in biology are devoted to spreading innovative methods in teaching biological science and making available more research-related curricula for undergraduates. In addition, investigators of all our individual research grants can ask for earmarked money to hire undergraduates so that they can pay them to work in their laboratories. This is especially important for equalizing access to research for undergraduates because in the US people from economically disadvantaged families typically have to spend the summer working to make money that helps them fund their education during the year. We try to enable investigators to pay students enough so that they do not have to have another job during the summer. We do that by giving students a stipend, and in addition we also support living expenses during these times of the year. By making such funds available to specifically involve undergraduates in research, another goal is to increase the recruitment and retention of students from backgrounds that are currently not very well represented in science.

Undergraduate research is also an explicit component of the Faculty Early Career Development (CAREER) Program, a class of awards reserved for assistant professors. As part of the application for these awards, the investigators need to have an explicit plan that enunciates clearly how they intend to combine teaching with research, and this especially focuses on the involvement of undergraduates in research. One of the means of doing this, for instance, is that the person can propose to offer a research-based course for upper-level undergraduates, with 15 or even 20 people involved in laboratory research. We will pay for the resources to make this possible. What people typically do is to break up the research in their grant so that it is possible for teams of undergraduates to do smaller projects. These are the kinds of ways that we are trying to encourage and spread research experiences for undergraduates.

*Günther K.H. Zupanc:* Can the recently established Research and Mentoring for Postbaccalaureates in Biological Sciences (RaMP) program be seen as an extension of the NSF’s commitment to support undergraduate research experiences?

*Evan Balaban:* Yes! This program supports the construction of mentoring and training networks for 3 cohorts of 8–12 postbaccalaureate graduates per year over the course of 3 years. The idea is to foster high-quality interactions of postbaccalaureate participants with faculty members and/or other diverse research mentors in a wide range of facilities. The goal is to improve collaboration, communication, professional development, and training opportunities for students who have just completed their university studies, and are at the threshold of research-related careers within and outside academia.

*Günther K.H. Zupanc:* Just to avoid any misunderstanding: For each of the programs you mentioned, it is the Principal Investigator (PI), and not the student, who can apply for funds supporting undergraduate research, correct?

*Evan Balaban:* Right, it is the PI. We do not currently have any fellowship program for undergraduate students. One of the reasons is that the number of applications would be enormous, and we just do not have the resources to administer something like this. So, we put this in the hands of the PIs. Typically, the way we prefer to do this is that when PIs apply for a grant, they build this money in. Let’s say you want to have four or five undergraduates working in your lab every year. You would then ask, as part of the original grant application, for the funds to support these students.

*Wolfgang Rössler:* Undergraduate support is not such a big topic in Germany, but PIs can apply for *studentische Hilfkräfte* (student research assistants) in grant applications. However, I have the feeling that there has been some change in recent years since an educational component must be added to the application—a justification for these positions, and not just a statement that students would do some simple work in the lab.

*Christoph Limbach:* Student assistants are one of numerous eligible budget categories, and PIs are free to choose this option. The idea is to get undergraduates interested and involved in research. This can be routine work in the lab, or more elaborate experimental work. It is not so much the educational perspective, but rather the justification by the research program that will be assessed by the reviewers—in other words, whether they believe that the project needs support by student assistants because it is laborious and demanding in terms of personnel. Once the grant has been approved and it turns out that more money is needed for student assistants, PIs are free to reallocate their budgets accordingly.

*Wolfgang Rössler:* I am not aware of any DFG funding program for which undergraduate students can apply on their own, correct?

*Christoph Limbach:* This is correct—applications for one’s own funding are only possible after successful completion of a doctorate.

### Finding and funding graduate student research positions: “match your interests with the specific research focus of individual universities, groups, and PIs”

*Günther K.H. Zupanc:* Which gets us to funding of PhD students. Most biology PhD students in the United States are supported by the research grants of their PIs. In addition, graduate students can apply for their own funding, for example through the Graduate Research Fellowship Program of the Directorate of Biological Sciences at NSF. This program is heavily subscribed. In 2020, NSF received 13,000 applications for these fellowships and made awards to 2000 of the student applicants (https://www.nsfgrfp.org/applicants/). So, the success rate is roughly 15%. First, please tell us more about this program, and second, can you give prospective applicants some advice on how to increase their chances for receiving a fellowship?

*Evan Balaban:* As you said, there are two routes. One route is what I call the indirect one for the student. That is, an investigator will ask in the research grant application for a certain number of doctoral positions on the project. If they have candidates, they can provide details in the grant application, but many of them do not. So, basically the PIs are the ones who choose PhD candidates.

The second route is student initiated. Students apply for a Graduate Research Fellowship. This research fellowship program exists NSF wide, not just in biology. Students can apply in their last year of university, or they can apply in each of their first few years after they get into graduate school.

*Günther K.H. Zupanc:* If the students apply in the last year of their undergraduate studies, do they need to know the laboratory they would like to join with their fellowship?

*Evan Balaban:* It helps if they know the lab. However, it is the area and the kind of research they are interested in pursuing that is important. We are not judging students on the qualifications of the person they are going to work with; rather, we are judging them on their previous record, and on the statement they prepare. This statement is brief, but it can be quite revealing about what training they intend to receive, and what they want to do with that training afterwards. So, it really helps them to be articulate and to have a realistic plan. And yes, overall, it is probably a good idea to have identified whom they want to work with before they submit one of these applications, because they can be more specific about their plans and training when they write their statement. But again, this is not so much about whom they are going to work with, as it is about what they have accomplished, the contents of their graduate plans, and what they are interested in doing with this training afterwards.

*Wolfgang Rössler:* Like in the United States, in Germany graduate students are primarily supported through the grants of their advisors. It seems that students themselves cannot apply for DFG funds. Is that correct?

*Christoph Limbach:* Yes, that is correct. As a general principle, eligibility to request DFG funding requires completion of a doctoral degree. However, the option to apply for PhD funds is available to PIs in all major funding programs. In addition, DFG runs a specific funding scheme for structured graduate programs known as the *Graduiertenkollegs* (Research Training Groups). Their emphasis is on the qualification of doctoral researchers within the framework of a focused research program and a structured training strategy. Students interested in a specific graduate program can apply directly to the Research Training Group.

I believe it is important for students planning their PhD (and the same is true for postdocs) to match their interests with the specific research focus of individual universities, groups, and PIs. In this regard, I would like to point to some web tools that may be of help. One such tool is DFG’s project database GEPRIS (http://gepris.dfg.de/), another one is DFG’s database of German Research Institutions (https://www.gerit.org/en/). I would also like to mention the Research in Germany initiative. For this initiative, German research and funding organizations have teamed up to provide information about Germany’s research system. The website (https://www.research-in-germany.org) is quite elaborate and provides ample information on the structural aspects of the German system, funding opportunities, and much more. On this website (https://www.research-in-germany.org/dam/jcr:4f90489a-4722-4e07-abfd-6682917b22fd/RiG_Neurosciences_October_2021.pdf), you can also find a booklet specifically describing the neuroscience community in Germany.

### Comparison of funding of postdoctoral research: United States versus Germany

*Günther K.H. Zupanc:* At the next career stage, the postdoctoral, funding seems to be rather straightforward in the United States. Most postdocs are funded through the research grants of their PIs.

*Evan Balaban:* They are. However, in the last few years the Biological Sciences Directorate of the NSF has started funding fellowships, independent from research grants and limited to a few target groups.

*Günther K.H. Zupanc:* Which are a rather unusual mix: people traditionally underrepresented in biology; researchers working on plant genome projects; and scientists investigating the rules of life governing interactions between genomes, environment, and phenotypes.

*Evan Balaban:* To understand this mix, it helps to know the historical context. In the late 1990s, plant genome research was lagging far behind genomic research in animals. NSF established a special program about 24 years ago, called the Plant Genome Research Program, which was specially mandated by Congress, to encourage more research in this area. They felt it was especially important to have postdoctoral fellowships available in this area. More recently, this fellowship program was expanded to include the two other categories you mentioned. The reason for having the third, broadly defined category is the desire to not exclude any particular area. Somewhat hidden between the lines is our goal to encourage people doing postdoctoral work that combines different scales of organization, and different areas in biology.

*Günther K.H. Zupanc:* At a more advanced postdoctoral level, is there anything offered by NSF like the Pathway to Independence Award, better known as the K99/R00 Award, at NIH?

*Evan Balaban:* We do not have an explicit program that bridges the postdoctoral stage with the first faculty-appointment stage. We pick up funding again at the junior faculty-career stage with our CAREER Program. These awards are explicitly for assistant professors only.

However, what we have instead is a certain flexibility in the definition of who can apply for a research grant. Universities define who is eligible to apply for a grant. They have the leeway to classify advanced postdocs as ‘research associates’ or ‘researchers,’ which makes these individuals eligible to be co-PIs on a grant. These senior postdocs can then transition from that research grant when they get their own independent position. Since there is this flexibility built into the system, we do not have a special program that bridges the two career stages.

*Wolfgang Rössler:* I think we are at an interesting point where we should look at differences between the two systems. In the US system, postdocs are appointed much earlier to their first faculty position, typically a tenure-track assistant professorship. In Germany, a tenure-track pathway does not exist in the broader sense. I think this is one reason why the postdoctoral funding offered by the DFG is rather complex, as it bridges the gap between postdoc, independent researcher, and faculty position. The funding schemes offered at this career stage range from the Walter Benjamin Program and one’s own position as part of a DFG grant all the way up to an Emmy Noether Research Group and a Heisenberg Fellowship. Would you agree that the marked differences in funding at this career stage are related to the fundamental differences between the two university systems?

*Christoph Limbach:* Yes, probably this is the reason. Tenure-track systems are emerging in Germany. It depends on the individual universities on how far their development towards a tenure-track system has advanced. In the traditional German system, researchers must cope with a certain period of insecurity until they secure a permanent position. They are employed on a temporary basis and depend on third-party funding. This challenge appears to be one of the reasons for the relatively high dropout rate of female researchers.

On the other hand, and as you mentioned, DFG offers ample opportunities for funding positions along the scientific career track. And there are additional funding organizations in the German system that also support researchers at these career stages.

*Wolfgang Rössler:* I agree that there is a great pool of funding opportunities provided by the DFG for postdocs. However, my feeling is that when PIs include a request for a postdoc in their *Einzelanträge* (individual research grant applications), these positions are not granted as readily as are PhD positions.

*Christoph Limbach:* The most important aspect in the review process is whether there is a good match between the project description and the requested research staff. We ask the reviewers and the Review Board to assess this match—and whether a postdoc is, indeed, needed to carry out the proposed work. One should keep in mind that the available budget is restricted. The Review Board, therefore, discusses funding priorities and may also recommend budget cuts if the justification is not fully convincing. As part of the revised budget, they may recommend funding a PhD position instead of a postdoc position. However, there is no automatism, and the budget is assessed specifically for each individual proposal.

As part of the research grant program, we also offer the funding module *Eigene Stelle zur eigenständigen Durchführung eines Forschungsprojektes* (‘Temporary Position as Principal Investigators’). This enables applicants to request funding at the postdoctoral level for their own positions as the project leaders. For early postdocs, we offer the Walter Benjamin Program. In addition, we fund postdoc positions in our research consortia programs (e.g., Collaborative Research Centers). Taken together, I think that there is quite a broad range of postdoctoral funding opportunities offered by the DFG.

*Wolfgang Rössler:* The Walter Benjamin Program is relatively new. Can you tell us more about it?

*Christoph Limbach:* The Walter Benjamin Program was established in 2019, and since then it has gradually replaced the DFG Fellowship Program. This new program enables researchers in the early postdoctoral phase to independently conduct their own research project at an institution of their choice. Postdocs from Germany can choose either to go abroad for up to two years on a fellowship basis, or to work on a research project at an institution in Germany, where they are employed on a temporary basis. The latter option is also open for ‘incoming’ researchers, i.e., postdocs from abroad who can apply for a postdoctoral position in a group in Germany. The Walter Benjamin program fosters mobility and thematic development. Funding entails the fellowship or salary plus various allowances. Further financial support, as well as training and mentoring, must be provided by the host institution.

*Wolfgang Rössler:* When you say, “incoming researchers,” do you mean foreign applicants or German applicants who live abroad?

*Christoph Limbach:* The program is open to early career postdocs from any country.

*Wolfgang Rössler:* Does the *Eigene Stelle* module have age or career-stage restrictions?

*Christoph Limbach:* There are no age or career-stage restrictions for the *Eigene Stelle* module. Nevertheless, this funding scheme preferentially targets early career researchers.

*Wolfgang Rössler:* When we talk to young scientists, we get the impression that they appreciate the availability of multiple funding programs, but one of their major concerns relates to predictability: Do I have a realistic chance of getting a professorship after a long period of insecurity? Should I quit after my PhD, or should I continue with a postdoc? For how long should I stay as a postdoc before I finally decide to remain in academia or not? Which career path would you recommend to a young postdoc?

*Christoph Limbach:* I do not think that there is a single best career path—in my opinion, it is important for postdocs to carefully analyze their specific situation and future perspectives, and then decide which step to take next. One thing that we consider important in the early postdoctoral phase is to show mobility, to change lab after the PhD, to get experience from a different scientific environment, and to broaden one’s individual expertise and outlook. This is why mobility is an important criterion in the Walter Benjamin Program.

At an advanced stage, an independent junior research group is likely to boost one’s research career. DFG’s funding scheme for independent junior research groups is the Emmy Noether program. Similar programs are available at other funding and research organizations. These programs are no doubt highly competitive. Our Emmy Noether program also has a relatively narrow time window; eligibility is restricted to a period of four years after completion of the doctorate. The program targets postdocs with high potential who have demonstrated outstanding achievements during their PhD and postdoctoral phases.

*Wolfgang Rössler:* Young scientists often voice their concern about the four-years-after-the-doctorate time window of the Emmy Noether program because it might conflict, for example, with family planning. Are there any flexibility criteria?

*Christoph Limbach:* In the Emmy Noether program, periods of childcare are taken into account so that the 4 years eligibility window can be extended.

Of course, not everyone can get an independent junior research group. On the other hand, this is not the only way to establish yourself in academic research. There are various options. We have already discussed several of them. Obviously, it is important to obtain good mentoring from colleagues and experienced researchers. And of course, we—my colleagues and myself—at the DFG Head Office are always available for consultation on funding opportunities. As we mentioned earlier, one option to secure funding for your own postdoc position is the *Eigene Stelle* module as part of an individual research grant. This module provides the salary for the applicant’s position as the PI of your own research project so that you can dedicate 100% of your time to the project.

*Wolfgang Rössler:* Let me return to the issue of predictability and security. As a young investigator, if I have done two great postdocs I might consider applying to the Emmy Noether Program to maximize my chances of getting a faculty position. Are there any data on the success rate of Emmy Noether research group leaders in securing a faculty appointment in Germany or abroad?

*Christoph Limbach:* In 2017, DFG published a comprehensive study on DFG Programs for Research Career Support. Among others, this study analyzed the career prospects of Emmy Noether-funded research scientists. Both an English summary of the study (https://www.dfg.de/download/pdf/dfg_im_profil/geschaeftsstelle/publikationen/infobriefe/ib02_2016_en.pdf) and the full report of the study (in German only; https://zenodo.org/record/1475864#.XZ310-TV5aQ%23.XZ310-TV5aQ) are available. This study shows that Emmy Noether group leaders were, during the time period analyzed, extremely successful. There was a very low dropout rate, and almost two-thirds of grantees were appointed to a faculty position within 7–8 years of the funding decision. Remarkably, 45% of the grant holders secured a professorship or equivalent position before the end of their Emmy Noether funding.

*Wolfgang Rössler:* How many of the successful candidates went on to become professors abroad?

*Christoph Limbach:* Roughly 12% of the Emmy Noether grantees moved to research positions abroad.

### Are grant applications from early career investigators evaluated differently?

*Günther K.H. Zupanc:* Let’s now discuss some aspects of funding at the independent-investigator level. On the cover sheet of the NSF proposal form, applicants are asked to indicate whether they are ‘beginning investigators.’ When I talk to colleagues, there seems to be quite some confusion about what this term means, and how the checking of this box affects the grant evaluation process.

*Evan Balaban:* This is good opportunity for clearing up this confusion—‘beginning investigator’ may be the least-well-chosen name given its technical meaning. To grant administrators, a beginning investigator is somebody who has not been a PI or co-PI on a federal research grant before. The only thing that checking this box does is that it allows the applicant to submit the same grant proposal (that is, the same research project) at the same time to multiple federal funding organizations. Then, if one or more agencies decides to fund it, the investigator can choose which funder to accept and proceed with the work. If an applicant is not a beginning investigator, they can only submit one project to one federal agency at a time.

Labeling yourself as a beginning investigator can have another advantage. At the NSF, funding decisions are made by program officers, not directly by a review panel (which gives program officers advice) or by rating numbers. Let’s say that you have two grant applications that appear to have equal merits. A program officer might consider the experience and the funding situation of the applicants. If one person is a beginning investigator, and the only research support he/she would have is this grant while the other person is an experienced researcher who currently has other funding, this may tip the funding decision in favor of the beginning investigator.

*Günther K.H. Zupanc:* Can you provide any information about the funding rate of beginning investigators compared to established investigators?

*Evan Balaban:* We have to factor out one confounding variable, which is that beginning investigators may choose not to consult with program officers or more senior colleagues before submitting a grant application, and consequently these applications may not be as well-prepared or well-written as those from non-beginning investigators. Poorly prepared proposals are usually designated by review panels as ‘Low Priority’ or ‘Not Competitive’. While we cannot release specific data about funding rates by career stage, the Biological Sciences Directorate does fund investigators throughout their career. And the funding rates published on our website for biology proposals are in general an accurate reflection for the different research areas funded by the Directorate for Biological Sciences, for example neuroethology or neural systems proposals.

*Wolfgang Rössler:* Does the DFG have something similar to the beginning investigator category? And if so, how does that affect the evaluation of the grant application?

*Christoph Limbach:* Applicants can, indeed, indicate that their grant proposal is an *Erstantrag* (first-time proposal). The major purpose is to highlight for the reviewers and the Review Board that this is a proposal from a less experienced early career investigator. However, the consequences are probably not as pronounced as they are widely assumed, simply because for any proposal the career stage of the PI is taken into account, no matter whether it is a first-time proposal or not. So, yes, the indication that the grant application is an *Erstantrag* helps to emphasize the fact that it is a proposal submitted by an early career scientist, but this does not result in a fundamental difference in the review process.

### Torn between science and family: how NSF and DFG address career-life balance

*Günther K.H. Zupanc:* Many investigators are torn between academic and family life. This conflict has a particularly adverse effect on the career progression of women. What support mechanisms does the NSF have in place to help grant and fellowship holders to better balance work and family life?

*Evan Balaban:* The NSF has been acutely aware of this problem, and the fact that career-life balance issues have particularly adverse effects on the careers of female scientists. We have been trying to address these issues through our Career-Life Balance Initiative. As part of this initiative, everybody can submit a supplemental funding request. Let’s say that your family responsibilities change, or that you have an adverse medical event, or somebody in your family has had such an event, and you need to step back a little bit from your research duties to take care of this situation. In such cases, the NSF would like you to apply for a Career-Life Balance Supplement. This equally applies to people with Graduate Research Fellowships, Postdoctoral Research Fellowships, or research grants. In each case, the idea is that you can ask for money (typically up to $50,000 in direct costs) to hire additional help in the lab to make up for the fact that you have to step back a little bit from research.

We also have ways of dealing with situations that do not require additional financial resources by granting no-cost extensions for research grants. Through this mechanism, we will give you extra time to complete the project. When you need both extra time and extra money, it is possible to ask both for an extension and for supplemental funding.

There is also a new resource that is tangentially connected to the Career-Life Balance Initiative. It is called the Mid-Career Advancement (MCA) program, and it targets investigators who are Associate Professors. This award funds your academic salary for a total of 6.5 months (which can be spread out over a period of up to 3 years) to allow yourself to either get training in somebody else’s lab or to initiate a new course of investigation in your own lab, in collaboration with other people who are able to train you in new things. It also provides support for this research activity and some support for your collaborators. The idea behind these awards is to help you take your career in a different direction than it is currently going.

*Wolfgang Rössler:* Let’s take a look at similar support mechanisms that the DFG has in place to facilitate career-life balance. My first specific question is whether an award period can be extended for researchers who take a leave of absence due to dependent-care responsibilities, including birth or adoption of a child?

*Christoph Limbach:* Yes, in such cases a no-cost extension of a grant can be requested. Under certain circumstances, it is also possible to temporarily halt the project. We have numerous options available to support researchers in such situations. We always try to identify the best solution for the individual project.

*Wolfgang Rössler:* Can the DFG funds be used to pay replacements of project personnel who take a leave of absence due to dependent-care responsibilities?

*Christoph Limbach:* Yes, that is possible. Besides the no-cost extension, the DFG can award additional funds for unforeseen project-related expenses, due to the absence of either the PI or the personnel funded through the project. For instance, if you need student research assistants to continue field work or to do routine lab work, we can award supplemental funds. Additionally, in 2019, the DFG introduced a family allowance as part of the Emmy Noether program. It can be used for covering the costs for the care of children and relatives while the PI or research staff attend conferences or are on research trips.

*Wolfgang Rössler:* A related question is whether institutions or organizations can use DFG funds for dependent-care expenses?

*Christoph Limbach:* There are various funding schemes for research consortia. As part of the project coordination in these schemes, it is possible to request a standard allowance for gender equality measures to facilitate promotion of equal opportunities and making jobs in science more family-friendly. While basic childcare must be provided by the host institutions, the allowance can be used to pay for costs outside the regular opening hours of day care facilities, e.g., when attending a seminar or conference on a weekend or late during the day. The allowance is available to all researchers who are part of the consortium. At a given location, gender-quality allowances awarded to several research consortia may be pooled for joint measures. In the latter case, we recommend consulting the DFG Head Office beforehand.

### Science has always benefited from the international exchange of ideas and researchers

*Günther K.H. Zupanc*: At the end of our conversation, we would like to discuss funding of international collaborations, including studying and working abroad. Science has always benefited from such exchanges of ideas and scientists. My first question goes to Evan: Can the Postdoctoral Research Fellowship in Biology, which we mentioned earlier, be used for working in a lab abroad, or is it restricted to carrying out research with a PI in the United States?

*Evan Balaban*: The answer to this question involves a technical difference between a research grant and a fellowship. Research grants fund the institutions where the research work is done. Fellowships are awarded to individuals and not institutions. If fellowship recipients are doing some work abroad as a planned part of their scientific program, this does not raise any difficulties.

*Günther K.H. Zupanc*: What about research collaborations between labs in different countries?

*Evan Balaban:* The NSF is open to collaborative projects involving US PIs and other investigators essentially from anywhere in the world, as long as there is a valid scientific reason why the project requires those specific foreign partners. The NSF can make funds available to pay for the parts of these projects carried out at foreign institutions, although we are unable to pay for studentships and salaries at foreign institutions. Typically, such international collaborative projects are supported by making the financial award to the institution in the United States where the PI is located. This institution then makes a subcontract award to the foreign institution. Through this mechanism, it would, for example, be possible to fund an investigator in Germany as a co-PI for their part of the research project. What’s needed is having a US investigator as the PI, and a German investigator as a co-PI who can provide expertise, techniques, resources, or research material that is essential for the project and which is not otherwise available to the US partner.

The NSF has also specific bilateral agreements with several countries. For example, all of the NSF grant-making directorates cooperate with the US-Israel Binational Science Foundation for joint funding of collaborative research programs. If a project is funded, the NSF pays for the US part and Israel pays for the Israeli part.

Recently, in November 2021, the NSF and the DFG signed a Memorandum of Understanding to facilitate support of collaborative basic research in biology and medicine at a molecular, subcellular, or cellular level, including theoretical approaches. Currently, this agreement does not include proposals encompassing tissues, organs, or whole animals, and currently does not include areas such as neuroethology. The NSF is unfortunately unable to comment about whether any extensions to this agreement are currently under consideration.

*Christoph Limbach:* Like the NSF, the DFG is very much open to team-up with international partner agencies for joint funding. We try to create opportunities where there seems to be a demand by the scientific community for cooperation in certain research areas. Thus, we are having numerous bi- and multilateral agreements for joint funding. In addition, DFG funding is available to project partners from the Middle East. Similarly, the DFG can provide funds that are passed on to project partners in developing countries, for example in Africa.

Obviously, the NSF is a very important international partner for us. We are extremely happy to be one of NSF’s international partners in the NeuroNex program, jointly funding international neuroscience research networks. As Evan mentioned, in November 2021 the DFG and the NSF established a bilateral Lead Agency Opportunity for funding of joint projects in molecular and cellular biology. So, once again, we are very much open to such activities if there is significant demand and support from the respective community. For us, it is important that the scientific scope of any of such activities is sufficiently broad, and that all grant proposals are processed at the same level of competition. By applying equal standards, the best proposals will be funded, no matter whether it is a national research grant or an international collaboration.

## Conclusions

The future of neuroethology, like of any other curiosity-driven research discipline, depends critically on governmental funding. While until the end of the 1990s major funding organizations in the United States explicitly supported such research, around the turn of the millennium a fundamental shift in funding policies took place at the NIH, triggered by a narrowing of its focus on disease-related biomedicine. Although the NSF, as the second major governmental funding agency, has upheld its commitment to support curiosity-driven biological research, our analysis indicates that the NSF has not been able to compensate for the lack of NIH funding. In fact, over the past decade approximately 10% fewer grant proposals in biological sciences (and likely in the field of neuroethology) have been funded by the NSF. In addition, the average inflation-adjusted grant amount awarded by the NSF has not changed over this period, although the costs for competitive research have increased far more rapidly than the consumer price index. This reduction in U.S. governmental funding is likely to be *the* major factor that has resulted in a continuous decrease in the research output of the American neuroethology community over the last 20 years.

Over the same time period, the German neuroethology community has benefited from continued support of curiosity-driven research by the DFG. As revealed by a PubMed search for articles with explicitly declared neuroethological content (indicated by the search term “neuroethol*” in all fields) and affiliation of their authors with German institutions, the number of such publications has roughly doubled between 2002 and 2021. Assuming that this sample is representative for neuroethological publications of authors with German affiliation as a whole, this would suggest a robust positive trend in research output of German neuroethologists over the past 2 decades.

Besides the importance of governmental funding for curiosity-driven research, we discussed with the representatives of the NSF and the DFG the support that their organizations especially provide for young investigators who would like to pursue a career in academia. This path is essentially the only option available for neuroethologists and, once entered, there is little latitude for switching to different careers in non-academic sectors of the job market. Further complications arise from the extremely long time that elapses between earning a PhD and securing a permanent position, and from the fact that this ‘qualification period’ coincides with the time that many young investigators start a family. In the United States, postdocs are typically appointed to tenure-track positions in neuroscience-related disciplines around 2–7 years after their PhDs. At most colleges and universities, tenure decisions are made after a probationary period of 7 years. However, when considering a faculty career, it is also important to know that the percentage of full-time faculty with tenure at degree-granting post-secondary institutions with tenure systems was just 44.6% in 2019–20, having continuously dropped from 56.2% in 1993–94 (National Center for Education Statistics 2019).

The DFG has developed excellent postdoctoral funding schemes, including funding for highly competitive junior research groups. While these programs are beneficial for young neuroethologists seeking permanent faculty employment in academia, potential candidates are concerned about the high level of uncertainty regarding their future career options. As this ‘qualification period’ may stretch over 6–10 years or even longer, candidates sometimes run into conflict with rather strict regulations for non-permanent employment in academia. Furthermore, only very few choices are left for late dropouts, irrespective of their excellent scientific qualifications. As a result, even some of the brightest PhD students in neuroethology are afraid of pursuing a career in academia. Consequently, it is important to improve the predictability of academic careers. A timely solution would be a substantial increase in early career tenure-track options—for instance, junior faculty positions for highly qualified candidates at an early career stage, with a reasonably good chance for being awarded tenure. Ideally, this should go hand in hand with the establishment of new governmental funding opportunities providing customized starting packages for successful candidates.

## Data Availability

All data used for preparation of Figs. 1, 2 are available from the authors upon request.
